# Optoelectronic Properties of Mixed Iodide–Bromide Perovskites from First-Principles Computational Modeling and Experiment

**DOI:** 10.1021/acs.jpclett.2c00938

**Published:** 2022-05-05

**Authors:** Yinan Chen, Silvia G. Motti, Robert D. J. Oliver, Adam D. Wright, Henry J. Snaith, Michael B. Johnston, Laura M. Herz, Marina R. Filip

**Affiliations:** † Department of Physics, University of Oxford, Clarendon Laboratory, OX1 3PU Oxford, U.K.; ‡ Institute for Advanced Study, Technical University of Munich, Lichtenbergstrasse 2a, D-85748 Garching, Germany

## Abstract

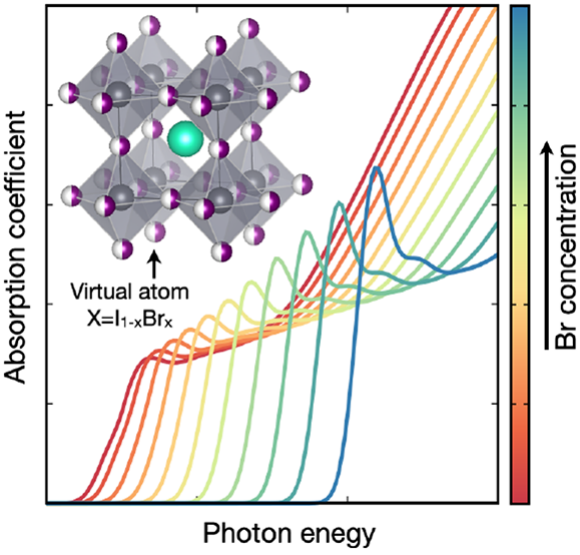

Halogen mixing in lead-halide perovskites is an effective route for tuning the band gap in light emission and multijunction solar cell applications. Here we report the effect of halogen mixing on the optoelectronic properties of lead-halide perovskites from theory and experiment. We applied the virtual crystal approximation within density functional theory, the *GW* approximation, and the Bethe–Salpeter equation to calculate structural, vibrational, and optoelectronic properties for a series of mixed halide perovskites. We separately perform spectroscopic measurements of these properties and analyze the impact of halogen mixing on quasiparticle band gaps, effective masses, absorption coefficients, charge-carrier mobilities, and exciton binding energies. Our joint theoretical–experimental study demonstrates that iodide–bromide mixed-halide perovskites can be modeled as homovalent alloys, and local structural distortions do not play a significant role for the properties of these mixed species. Our study outlines a general theoretical–experimental framework for future investigations of novel chemically mixed systems.

Perovskite solar cells have shown an unprecedented growth in power conversion efficiency, from less than 5%^1^ in 2009 to current certified records exceeding 25%. Rapid progress has been due to continuous development of both single-junction^2,3^ and tandem solar cell architectures^4,5^ as well as thin-film deposition techniques which permit precise and scalable control of the active material.^6,7^ More recently, the mixing of the organic/inorganic cations,^8,9^ metals,^5^ and/or halogens^10^ in the perovskite crystal structure has been highlighted as a versatile route to not only tune the optical absorption edge but also to improve the stability of metal halide perovskites. However, understanding the fundamental structural and optoelectronic properties of perovskites has been challenging from both a theoretical and an experimental point of view not only because of the structural complexity and disorder typically associated with chemical mixing in solids but also due to the segregation of halogen species within mixed-halide perovskite samples under above band gap illumination.^11^

Metal halide perovskites, with the standard chemical formula ABX_3_, are semiconductors with direct band gaps which span the visible range depending on their chemical composition.^12,13^ Their electronic band structure consists of a predominant contribution from the divalent B-site metal and the X-site halogen anions at the band edges,^14^ while A-site monovalent cation states tend to be located far below or above the valence band top and conduction band bottom, respectively.^14^ As a result, the band gap of lead-halide perovskites can be directly tuned over more than 1 eV by chemical substitution of halogen species, blue-shifting with decreasing size of the halogen anion.^15^ Similarly, replacement of Pb by Sn reduces the band gap by more than 400 meV.^16−18^ By contrast, A-site cations indirectly influence the electronic properties by inducing steric effects on the inorganic BX_6_ octahedra;^19^ these yield a band gap tunability range of more than 300 meV, red-shifting as the size of the cation increases.^19−21^

While metal-halide perovskite band gaps can be finely tuned via chemical mixing, the dependence on mixing composition is not strictly linear and/or monotonic, an effect known as “band gap bowing”.^22,23^ The most illustrative examples among halide perovskites are the MAPb_1–*x*_Sn_*x*_I_3_ and FAPb_1–*x*_Sn_*x*_I_3_ series, with band gap minima obtained for 25% and 50% Pb, respectively.^5,24^ Previous computational studies have focused on understanding the band gap bowing behavior on the mixed Pb/Sn perovskites. Reference (25) concludes from its density functional theory (DFT)^26^ and *GW*(27) calculations that the nonlinear trend in the band gap emerges from the nonlinear mixing of Pb and Sn orbitals in the band edges while other studies focus on steric effects, spin–orbit coupling, and local structural distortions.^5,28^ Overall, these analyses indicate that mixed-metal halide perovskites may not be suitably modeled as uniform alloys, and other secondary effects must be carefully accounted for. By contrast, mixed-halide perovskites exhibit only a slight bowing and a monotonic dependence of the band gap on the halide composition.^21,29−31^

Despite the remarkable advances in experimental studies of mixed-halide perovskites, most theoretical studies to date have been focusing on understanding perovskites with a single-atom occupancy per site. Optical and electronic structure properties of lead-halide perovskites have been studied extensively by using first-principles computational modeling techniques within DFT as well as the *GW* approximation and the Bethe–Salpeter equation (BSE).^32,33^ Electronic band structures of Pb- and Sn-based halide perovskites exhibit strong relativistic effects, with spin–orbit coupling dramatically changing the topology of the conduction band edge and reducing the band gap by more than 1 eV.^34,35^ In addition, *GW* calculations of the quasiparticle band structure of halide perovskites highlight the sensitivity of computed band gaps on the underlying DFT starting point,^36−38^ implementation of spin–orbit coupling,^17,38^ and the importance of thermal fluctuations in high-temperature phases of halide perovskites.^39^ Optical absorption spectra computed within the *GW*+BSE framework accurately resolve the excitonic features measured for halide perovskites,^40^ and calculations of exciton binding energies reveal that phonon effects play an important role in the physics of electron–hole interactions in these systems.^18,40,41^ Furthermore, calculations of electron–phonon interactions from first-principles underline the origins of photoluminescence broadening,^42^ polaronic mass enhancement,^43^ and charge-carrier transport.^44^

Mixed-halide perovskites have so far been studied within DFT using large supercells.^45−49^ However, this approach generally requires a large number of calculations on very large systems to accurately describe compositional disorder and becomes prohibitive for first-principles methods within and beyond DFT. Alternatively, the virtual crystal approximation (VCA)^50^ is an efficient approach used to understand structural, vibrational, and optoelectronic properties of disordered homovalent alloys.^50,51^ This method relies on the premise that chemically mixed species with the same electronic configuration behave as an interpolation between the pristine species and can be replaced by so-called “virtual” atoms that are uniformly distributed in the cell;^50^ therefore, the average primitive unit cell can be used to perform standard electronic structure calculations. However, limitations of VCA are expected if the nonlinear orbital mixing at the band edges or local structural distortions due to the rearrangement of the lattice around different ions have considerate first-order contributions to optoelectronic properties. These effects have been closely connected with significant band gap bowing in some semiconductor alloys.^5,25,52,53^ So far, the VCA has been used to understand structural and electronic properties of mixed-halide perovskites by using semilocal DFT and hybrid functionals.^54,55^ To the best of our knowledge, the VCA has yet to be used in conjunction with the *GW*+BSE framework for this family of materials. Given the central role that methods beyond DFT have played thus far in predicting excited state properties of pristine perovskites, as well as designing new materials for photovoltaic devices,^56−58^ it is important to establish an equally reliable framework to understand excited-state properties of chemically mixed-halide perovskites.

In this work, we present a study of the structural, vibrational, electronic, optical, and transport properties for a full series of mixed I/Br perovskites in the cubic phase by using a joint theoretical–experimental approach. The central question of our study is whether the model of uniform homovalent alloys is sufficiently accurate to capture the physics underpinning the key properties listed above. To establish this, we rely on the premise that significant discrepancies between trends computed via VCA and experiment can be associated with structural distortions or clustering of chemical species in different regions of the film and would therefore void the assumption of uniformity. Mixed iodide–bromide perovskites within a certain Br composition range are known to exhibit halide segregation, where iodide-rich and bromide-rich phases build up in separate regions of the film under specific illumination conditions. For example, MAPb(Br_*x*_I_1–*x*_)_3_ are known to segregate under relatively low illumination for Br concentration between 0.2 and 1.^11,59,60^ In contrast, mixed A-cation FA_0.83_Cs_0.17_Pb(Br_*x*_I_1–*x*_)_3_ perovskites have shown the most robust stability against halide segregation for a comparable Br concentration range.^10,61^ Because smooth trends in optoelectronic properties have been previously associated with mixing uniformity,^62,63^ we chose to focus the experimental study of FA_0.83_Cs_0.17_Pb(Br_*x*_I_1–*x*_)_3_. Samples were carefully synthesized to minimize inhomogeneities, as detailed in the Supporting Information, and all care was taken to ensure that optoelectronic properties reflect those of unsegregated materials. Computationally, we model this system starting from two fundamental assumptions: we simulate disordered chemical mixing of I/Br anions using the VCA (thereby assuming a uniform distribution of halogen ions and neglecting local structural distortions of octahedra), and we replace all FA cations with Cs. The former assumption is probed in the following by explicit comparison of trends in the lattice parameters, phonon frequencies, quasiparticle band gaps, exciton binding energies, and charge-carrier mobilities obtained from both computational modeling and experiment. While the absence of FA cations in our calculations may neglect some secondary effects on the band structure due to structural distortions,^64^ these are reasonably expected to contribute as a systematic uncertainty to our calculations, given that all measurements are performed on films with the same FA/Cs concentration. Replacement of FA cations with Cs atoms limits spurious static structural distortions due to the orientationally disordered FA cation and is justified by prior studies which already established that organic cations do not significantly contribute to optical or transport properties.^65,66^

First, we focus on structural and vibrational properties. In Figure 1a, we show a comparison of the lattice parameters obtained from calculation and experiment reported in ref (10) for the full series of mixed I/Br compositions (see the Supporting Information for computational details). Lattice parameter calculations based on the Perdew–Burke–Ernzerhof parametrization of the exchange correlation potential (DFT-PBE)^68^ exhibit the best agreement with experimental measurements; this result is due to a spurious cancellation of errors between the tendency of PBE to overestimate lattice parameters^69^ and the expectation to underestimate lattice constants by performing structural optimizations in the absence of FA cations. On the basis of the close agreement of computed lattice parameters with measurements recently reported in ref (10), we choose the PBE functional for all subsequent calculations presented in this work. Both computed and measured lattice constants follow Vegard’s law and display a nearly linear dependence on the Br concentration.^67^

**Figure 1:**
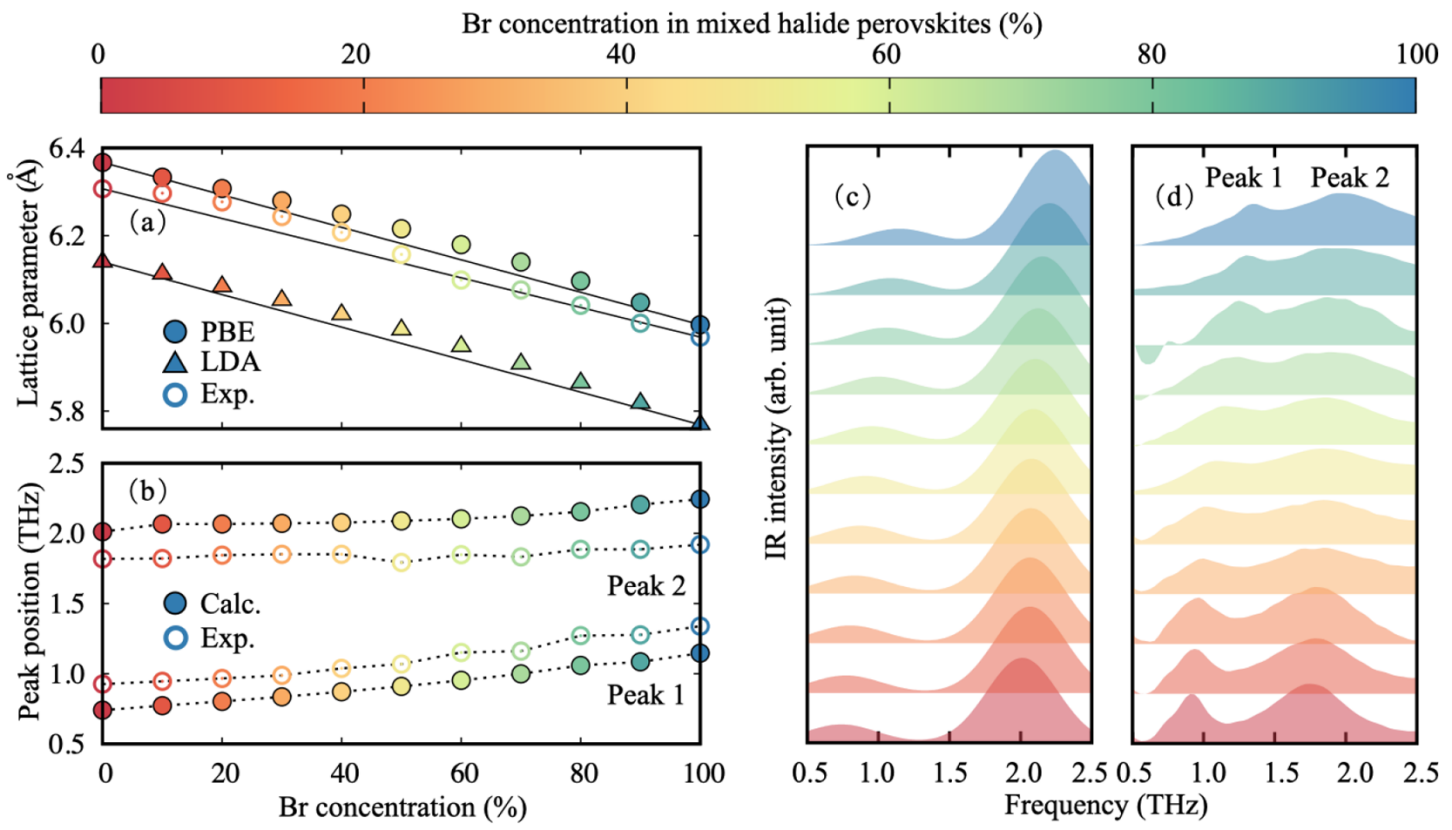
(a) Comparison between optimized lattice parameters computed within DFT/PBE (filled circles), DFT/LDA (filled triangles), and from measurements (open circles). The measured lattice parameters are for FA_0.83_Cs_0.17_Pb(Br_*x*_I_1–*x*_)_3_, extracted from ref (10). The continuous lines represent the linear dependence of lattice parameters according to Vegard’s law,^67^*a*_Br_*x*_I_1–*x*__ = (1 – *x*)*a*_I_ + *xa*_Br_, where *a*_Br_*x*_I_1–*x*__ is the lattice parameter of the mixed halide, *x* is the concentration of Br, and *a*_I_ and *a*_Br_ are the corresponding lattice parameters of the pure iodide and bromide compound obtained from DFT/LDA and DFT/PBE calculations or experiments. (b) Frequency of the first and second peak in the terahertz (THz) absorption spectra calculated within DFPT (filled circles) and measured experimentally (open circles) for a full series of mixed-halide perovskites. The dashed lines are guides to the eye. (c, d) Comparison between the calculated (c) and measured (d) THz absorption spectra of halide perovskite series. Computed spectra include an empirical Gaussian broadening corresponding to a full width at half-maximum (FWHM) of 0.6 THz, chosen so as to reproduce experimental spectra. Both computed and experimental spectra are normalized. The color of each data point and line corresponds to the different Br concentrations as indicated by the color bar.

To understand vibrational properties of this mixed series, we employ density functional perturbation theory (DFPT)^70^ using the VCA as described in the Supporting Information. We calculate 15 zone center phonon modes across the entire mixed-halide series (in decreasing order of their energy): two groups of triply degenerate IR-active modes, one group of triply degenerate nonpolar modes, three acoustic modes, and three modes with imaginary phonon frequencies. The latter are directly linked with anharmonicity and the structural instability of the cubic perovskite structure in the absence of temperature effects.^71^ Calculated and measured terahertz (THz) spectra exhibit two main IR-active peaks in the 0.5–2.5 THz frequency region. As shown in Figure 1, the calculated positions of these peaks are in good agreement with measurements, with differences of less than ±0.3 THz (∼±1 meV) found across the entire range of concentrations. Slight discrepancies in the calculated positions and spectral weights of IR-active peaks can be assigned to multiple factors, including the absence of the FA cation in our atomistic models, the small mismatch between computed and measured lattice parameters, and the absence of temperature effects. Importantly, both IR-active mode frequencies display the same dependence trend on Br concentration as in experiment, in agreement with our assumption of uniformity.

Next, we turn our attention to the electronic properties. In Figure 2a, we show a comparison of quasiparticle band gaps for the CsPb(Br_*x*_I_1–*x*_)_3_ series calculated within the one-shot *G*_0_*W*_0_ approximation, including spin–orbit coupling, as implemented in the BerkeleyGW code,^72^ by using Kohn–Sham eigenvalues and eigenfunctions calculated within DFT-PBE (hereafter termed *G*_0_*W*_0_@PBE), as implemented in the Quantum Espresso code^73^ (computational setup along with detailed convergence tests are found in the Supporting Information). We compare our calculations against experimental quasiparticle band gaps extracted from optical absorption spectra using the Elliott model^74^ (as discussed in the Supporting Information and in ref (75)). Our calculated band gaps consistently underestimate the experiment by ∼0.9–1 eV, while accurately reproducing the measured trend with respect to the Br concentration. This large quantitative discrepancy has two fundamental origins. First, the *G*_0_*W*_0_ framework generally yields quasiparticle band gaps which are strongly dependent on the mean-field starting point;^37,76^ this can in principle be addressed by using suitable hybrid functional starting points^76^ or by implementing self-consistency,^36,38,39^ both highly computationally demanding techniques. In particular, self-consistency has been shown to blue-shift quasiparticle band gaps by 0.4 and 0.6 eV for lead-iodide and lead-bromide perovskites, respectively.^39^ Second, quasiparticle band gaps computed for the high-temperature cubic perovskite phase carry a systematic underestimation due to the absence of thermal effects which blue-shift the quasiparticle band gap of cubic CsPbI_3_ and CsPbBr_3_ by 0.7 and 0.5 eV, respectively.^39^ The systematic underestimation of our computed quasiparticle band gaps is thus fully accounted for by these two contributions. To correct it, we apply a rigid shift of 1.1 eV across all series (based on ref (39)) and hereafter refer to this shift as a “thermal correction”. In addition to a band gap blue-shift, we note that an increased concentration of Br also leads to an increase in the valence bandwidth by up to 0.5 eV and a subtle decrease in the curvature of the valence and conduction band edges, as shown in the quasiparticle band structure (Figure 2b), which indicates a change in charge-carrier effective masses.

**Figure 2:**
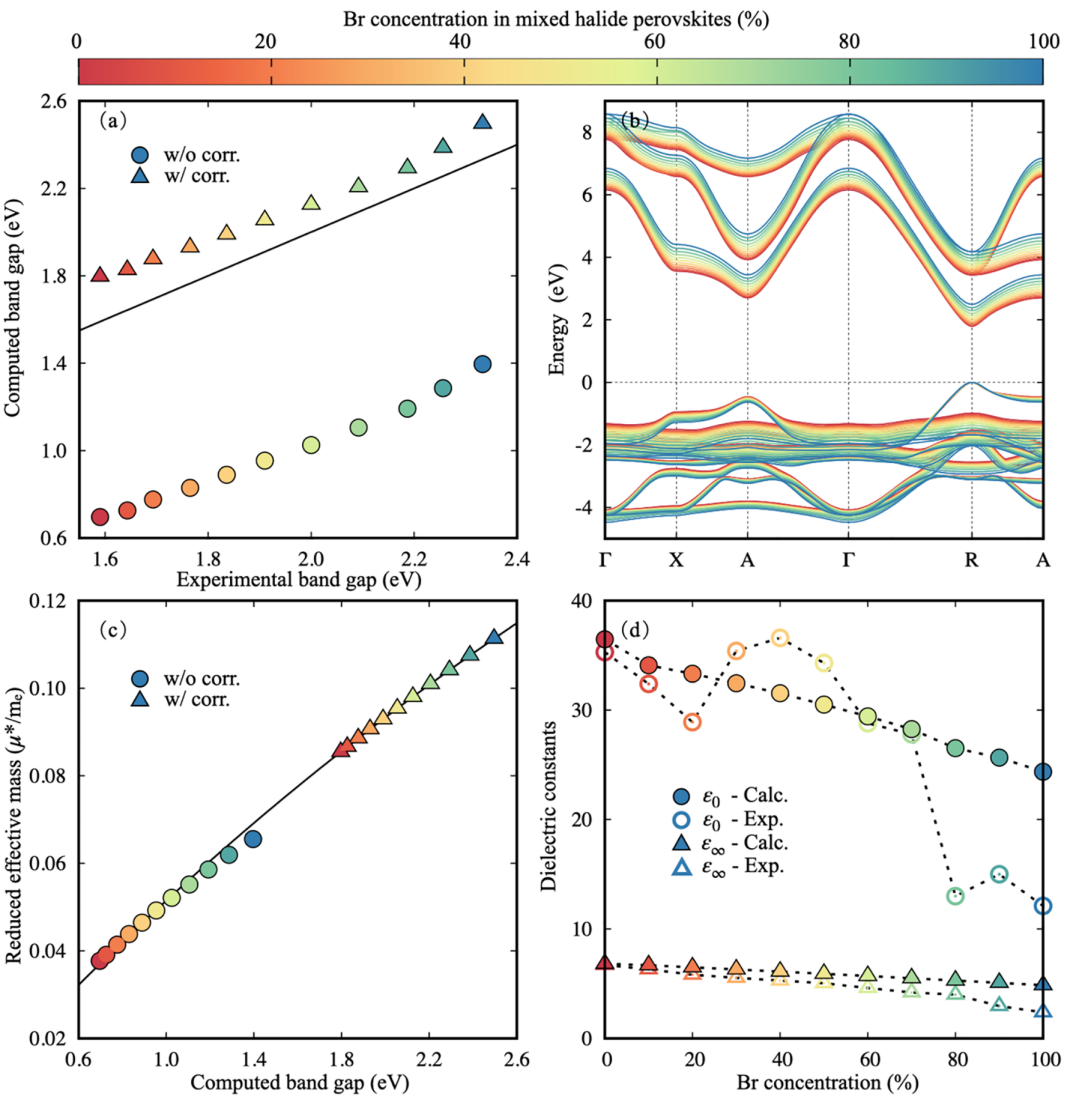
(a) Calculated *G*_0_*W*_0_ quasiparticle band gaps with (triangles) and without (circles) thermal corrections plotted against measured band gaps extracted from optical absorption spectra measured for FA_0.83_Cs_0.17_Pb(Br_*x*_I_1–*x*_)_3_. The continuous black line corresponds to the line of perfect agreement between theory and experiment. (b) Quasiparticle band structures calculated from *G*_0_*W*_0_@PBE including thermal corrections, aligned to the top of the valence band. (c) Computed reduced effective masses (μ*) as a function of band gaps (*E*_g_) with and without thermal corrections (triangles and circles, respectively). The black line corresponds to the expression *m*_e_/μ* = 2 + 17.43/*E*_g_ (where *m*_e_ is the electron rest mass), obtained by fitting uncorrected effective masses using **k**·**p** perturbation theory. Effective masses with thermal corrections are extrapolated by using this expression. (d) Comparison of low- and high-frequency dielectric constants calculated from DFPT for CsPb(Br_*x*_I_1–*x*_)_3_ and measured experimentally for FA_0.83_Cs_0.17_Pb(Br_*x*_I_1–*x*_)_3_. The dashed black line is a guide to the eye. All data points are color coded according to the concentration of Br, as indicated by the color bar.

In Figure 2c and Figure S3 we calculate the charge-carrier effective masses of mixed-halide perovskites (see Supporting Information for details). Reduced effective masses calculated within standard *G*_0_*W*_0_@PBE are underestimated with respect to tabulated experimental values in the literature^77^ by more than 50%, consistent with the quasiparticle band gap underestimation described above and in agreement with past calculations.^44^ At the same time, effective masses closely follow the dependence on the band gap predicted by **k**·**p** perturbation theory,^78,79^ exhibiting a linear dependence of the inverse effective masses against inverse quasiparticle band gaps with a slope of 17.4 eV. This trend is in excellent agreement with the fit performed for measured effective masses in ref (77), reporting a slope of 17.3 eV. Here, we use the **k**·**p** perturbation theory to extrapolate to effective masses which include the thermal correction of computed band gaps described above (see Figure 2c and Figure S3), which will be used in subsequent analyses.

In Figure 2d, we complete the analysis of the electronic properties with a comparison between high-frequency (ε_∞_) and low-frequency (ε_0_) dielectric constants obtained from theory and experiment. High-frequency dielectric constants computed within the random-phase approximation (RPA)^80,81^ exhibit a very good agreement with experiment across the mixed-halide series; this is consistent with prior calculations of dielectric constants in the literature.^40^ For low-frequency dielectric constants, we note that the range of computed and measured values are largely in good agreement. However, experimental values show some nonsystematic discrepancies. These discrepancies mostly arise from experimental uncertainties because of variations in substrate and film thickness, accuracy of fits used to extract the values from the complex dark conductivity spectra, and general signal-to-noise of acquired data.

In the final part of our analysis, we focus on optical and transport properties. Computed and measured optical absorption spectra (see the Supporting Information for details) shown in Figures 3a and 3b, respectively, exhibit absorption coefficients in close qualitative agreement, with very similar line shapes across the entire I/Br series: an excitonic resonance at the onset of absorption, followed by a flat plateau and a sharp rise associated with the second lowest direct optical transition.^75^ Experimental optical absorption spectra at concentrations of 80% and above have the greatest contribution from disorder and scattering factors, which in turn lead to a departure from the smooth dependence of the measured exciton binding energies on the mixing concentration. Overall, in Figure 3c we show a very good agreement between measured and computed exciton binding energies, within the *G*_0_*W*_0_+BSE framework (see the Supporting Information for computational details and convergence). Importantly, we note that the quantitative agreement between computed and measured exciton binding energies is due to the cancellation of two separate effects that are not captured in our standard *G*_0_*W*_0_+BSE calculations. First, computed reduced effective masses (and therefore binding energies) are underestimated by more than a factor of 2 in the absence of thermal corrections, as discussed above. (The effect of thermal corrections to the exciton binding energy is illustrated by the triangles in the inset of Figure 3c.) Second, without including of phonon screening and polaronic interference effects,^40,83^ the *GW*+BSE framework has been shown to overestimate exciton binding energies for lead halide perovskites by up to a factor of 3. (Phonon screening effects on the exciton binding energy are illustrated by the squares in the inset of Figure 3c.) These contributions cancel each other out to yield the close agreement with experiment shown in the main panel of Figure 3c. However, despite these systematic error cancellations, the dependence of exciton binding energy on Br concentration follows a consistent trend, regardless of the level of theory used in computations.

**Figure 3:**
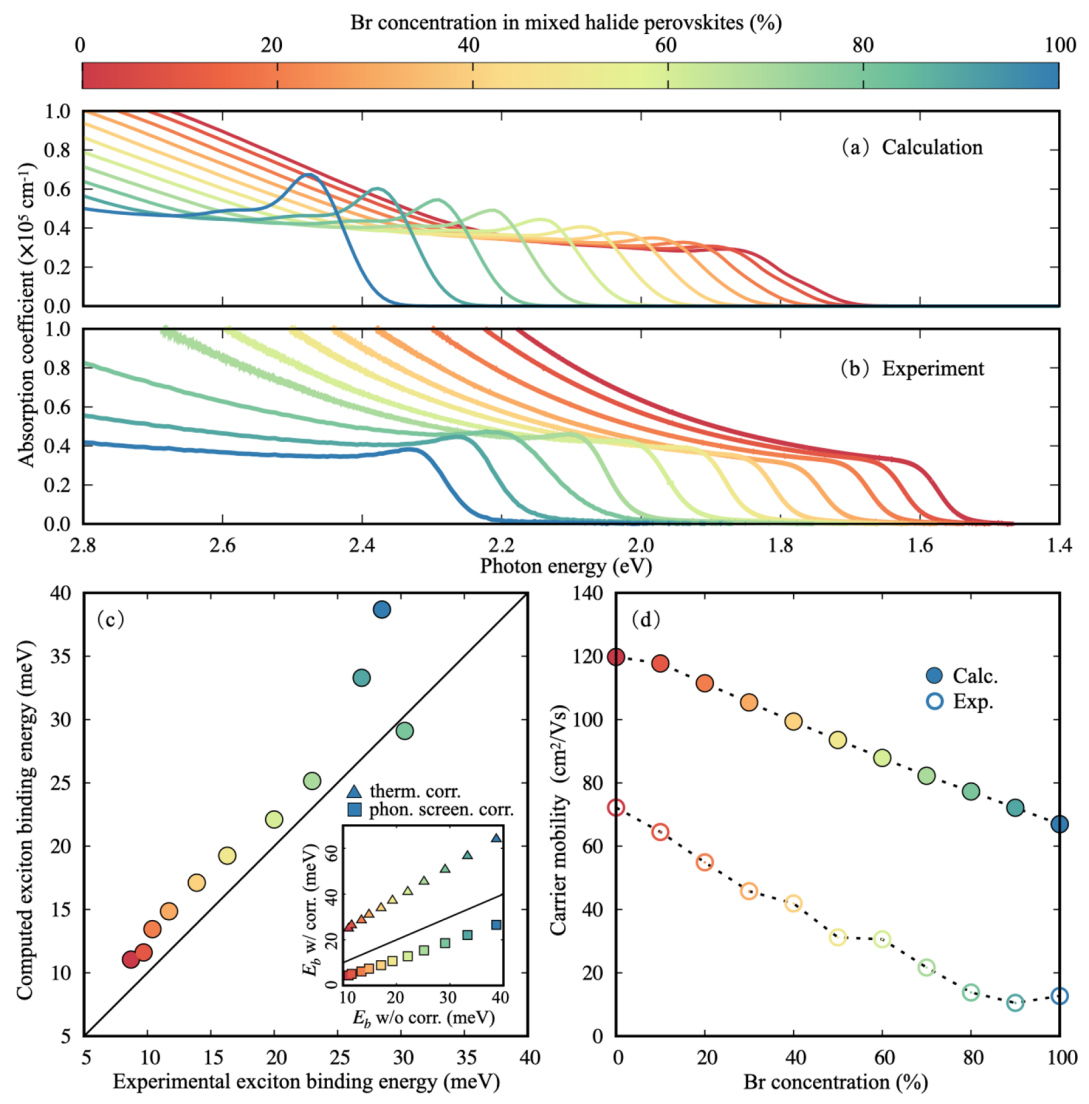
Calculated (a) and measured (b) optical absorption spectra for CsPb(Br_*x*_I_1–*x*_)_3_ and FA_0.83_Cs_0.17_Pb(Br_*x*_I_1–*x*_)_3_, respectively, across the entire I/Br series. (c) Computed *G*_0_*W*_0_+BSE exciton binding energies plotted against measured exciton binding energies extracted from optical absorption spectra by using the Elliot model,^74^ as described in the Supporting Information; the continuous black line represents the line of perfect agreement between calculations and experiment. The inset illustrates the contributions to the calculated exciton binding energies from thermally corrected reduced effective masses (triangles) and phonon screening effects^40^ (squares), as calculated via the hydrogenic model,^82^ which yields exciton binding energies in very close agreement with *ab initio* values (see Figure S5). The continuous black line corresponds to the line of null correction. (d) Electron–hole sum mobilities calculated by using the model proposed by ref (44) (filled circles) and measured experimentally (open circles). The dashed lines are guides to the eye. In all plots, the data point colors follow the concentration of Br, indicated by the color bar.

Finally, we calculate charge-carrier mobilities by using the model introduced in ref (44), whereby electronic bands are approximated as isotropic and parabolic and charge carriers are approximated to couple to a single LO phonon (using parameters extracted from our calculations; see Table S1 for details). In Figure 3d, we compare computed and experimental electron–hole sum mobilities across the I/Br mixed-halide series. The measured THz mobility probes the material in a relatively short range (tens of nanometers, shorter than other methods^65^). Therefore, it reflects more of the intrinsic properties of the lattice and is less affected by the morphology and other artifacts, yielding a clean and smooth experimental trend without any bending or other nonmonotonic effects. Computed electron–hole sum mobilities consistently overestimate experimental ones by almost a factor of 2 throughout the series, which we assign to scattering mechanisms other than electron–phonon coupling with a single LO phonon (e.g., scattering with other phonons and/or defects) not being included in our calculations.^44^ Despite these discrepancies, calculated charge-carrier mobilities display the same trend and order of magnitude as those measured, validating once again the assumption of a uniform distribution of halogen ions in these perovskites and confirming that long-range electron–phonon interactions dominate charge-carrier transport in halide perovskites across this series, fully consistent with prior studies of MAPbI_3_.^42,44^

In conclusion, we have presented a systematic study of the optoelectronic properties of mixed-halide perovskites from theory and experiment. Starting from the premise that the mixed halogen composition is uniformly distributed within the perovskite thin film, we have modeled the optoelectronic properties of mixed-halide perovskites by employing the VCA within state-of-the-art first-principles techniques including DFT, DFPT, and *G*_0_*W*_0_+BSE. We calculated and compared directly with experimental measurements a broad range of structural, vibrational, electronic, optical, and transport properties, such as lattice parameters, THz and UV–vis optical absorption spectra, dielectric constants, quasiparticle band gaps, exciton binding energies, and charge-carrier mobilities. A recurrent observation of the comparative analyses of theoretical and experimental results obtained in this work is that the VCA can accurately reproduce measured dependence trends of both ground- and excited-state properties. Consequently, these results emphasize that the tunability achieved via chemical mixing for key quantities such as band gaps or charge-carrier mobilities is not due to spurious inhomogeneities in samples or disorder-induced effects (which may be hard to control), but it is instead achieved by tuning the energetics of the halogen site and the unit cell volume via a uniform mixing of Br and I. Furthermore, our results clearly delineate the ideal behavior of mixed-halide perovskites, providing a template against which phenomena such as degradation, halide segregation, structural disorder, or sample inhomogeneity may be easily identified simply by analyzing trends in one of the several properties discussed here. Given the importance of homovalent alloyed perovskites in the continuous development of efficient photovoltaic devices, and in particular tandem cell architectures, the framework presented here can be used as a general and reliable approach to understanding chemistry–property relationships in these complex systems.

## Supporting Information Available

Computational setup for DFT and *GW*+BSE methods, experimental details including sample preparation and spectroscopic measurements, and additional references^84−92^ (PDF)
