# Supplementary material for: Optoelectronic Properties of Mixed Iodide–Bromide Perovskites from First-Principles Computational Modeling and Experiment
Source: J Phys Chem Lett. 2022 May 5;13(18):4184–92. doi: 10.1021/acs.jpclett.2c00938 (PMC9109221; doi:10.1021/acs.jpclett.2c00938)
Supplement: Supplementary file 1 — jz2c00938_si_001.pdf [file jz2c00938_si_001.pdf]

Supporting Information
Optoelectronic Properties of Mixed Iodide–Bromide Perovskites from First-Principles Computational Modeling and Experiment

Yinan Chen,[†] Silvia G. Motti,[†] Robert D. J. Oliver,[†] Adam D. Wright,[†] Henry J. Snaith,[†] Michael B. Johnston,[†] Laura M. Herz,[†,‡] and Marina R. Filip[*,†]

†*Department of Physics, University of Oxford, Clarendon Laboratory, OX1 3PU Oxford, U.K.*

‡*Institute for Advanced Study, Technical University of Munich, Lichtenbergstrasse 2a, D-85748 Garching, Germany*

E-mail: marina.filip@physics.ox.ac.uk

Computational Details

Ground state calculations

In this work, we carried out first principles calculations based on DFT[1] using plane-wave pseudopotentials methods as implemented in the Quantum Espresso package (version 6.5).[2,3] We employ fully-relativistic PBE norm-conserving pseudopotentials from the Pseudo Dojo repository,[4,5] with the following valence electron configurations: Cs $5s^2 5p^6 6s^1$, Pb $5d^{10} 6s^2 6p^2$, I $5s^2 5p^5$ and Br $4s^2 4p^5$. The pseudopotentials of virtual halogen atoms are constructed using the virtual_v2.x utility in the Quantum Espresso (version 6.5) package.[3,6] We use a kinetic

energy cutoff of 60 Ry and sample the Brillouin zone using a Γ centered $6\times6\times6$ Monkhorst-Pack mesh. The atomic positions and the lattice parameters are fully optimized until the energy was less than 10^{-7} eV and the absolute value of force on the atom was less than 0.01 eV/Å. All structural optimizations are performed using a $6\times6\times6$ **k**-point grid and without including spin-orbit interactions. In all other calculations reported throughout this work, spin-orbit coupling is fully accounted for.

We employ Density Functional Perturbation Theory (DFPT)[7] as implemented in the Quantum Espresso code to calculate vibrational properties of mixed halide systems. We calculate the normal vibrational modes within the harmonic approximation at the Brillouin zone center, using a dense grid of $12 \times 12 \times 12$ for the ground state charge density. This setup was chosen in order to achieve a converged dielectric permittivity for $CsPbI_3$ and $CsPbBr_3$, as shown in Figure S1a. The IR absorption spectra shown in Figure 1 of the main manuscript are calculated as in Ref. 8, where we use a Gaussian broadening corresponding to a full width at half-maximum (FWHM) of 0.6 THz, in order to match the experimental line shape. For all mixed species we calculate the atomic masses of virtual atoms by linearly interpolating between Br and I as we change the mixing ratio. In all DFPT calculations we apply the acoustic sum rule to the interatomic force constants.

Quasiparticle band structure calculations

We calculate quasiparticle eigenvalues within the GW approximation,[9] as implemented in the BerkeleyGW package[10] as, $E_{n\mathbf{k}}^{\mathrm{QP}} = \epsilon_{n\mathbf{k}} + Z\left(\epsilon_{n\mathbf{k}}\right) \langle n\mathbf{k} \left| \Sigma\left(\epsilon_{n\mathbf{k}}\right) - V_{\mathrm{xc}} \right| n\mathbf{k} \rangle$, where $\epsilon_{n\mathbf{k}}$ are the mean-field DFT-PBE eigenvalues, $\Sigma(\omega)$ is the electron self energy operator, V_{xc} is the exchange-correlation potential, and $Z(\omega)$ is the quasiparticle renormalization factor expressed as $Z(\omega) = [1 - \mathrm{Re}(\partial\Sigma/\partial\omega)]^{-1}$.

In all cases we perform one shot G_0W_0 calculations with a DPT-PBE starting point including spin orbit coupling. Within the G_0W_0 approximation, the electronic self energy Σ is approximated as the convolution of screened Coulomb interaction W_0 and single particle

Green's function G_0, written schematically as $\Sigma = iG_0W_0$. The single particle Green's function is calculated starting from DFT as, $G_0(\mathbf{r}, \mathbf{r}'; \omega) = \sum_{n\mathbf{k}} \frac{\psi_{n\mathbf{k}}(\mathbf{r})\psi^*_{n\mathbf{k}}(\mathbf{r}')}{\omega - \epsilon_{n\mathbf{k}} - i\eta}$, where the summation runs over occupied and unoccupied states, $\psi_{n\mathbf{k}}(\mathbf{r})$ is the DFT wave function corresponding to the energy eigenvalue $\epsilon_{n\mathbf{k}}$ and η is an infinitesimally small constant, positive for occupied states and negative for unoccupied states. The screened Coulomb interaction is given by the expression, $W_0(\mathbf{r}, \mathbf{r}'; \omega) = \varepsilon^{-1}(\mathbf{r}, \mathbf{r}'; \omega)\, v(\mathbf{r}, \mathbf{r}')$, where $v(\mathbf{r}, \mathbf{r}') = 1/|\mathbf{r} - \mathbf{r}'|$ is the bare Coulomb potential and $\varepsilon(\mathbf{r}, \mathbf{r}'; \omega)$ is the dielectric function. We model the frequency-dependent dielectric function via the Godby-Needs plasmon-pole model.[11]

We converge the dielectric constant and quasiparticle band gap with respect to the density of the $\mathbf{k}$-point mesh, as shown in Figure S1a and b, and find that the quasiparticle band gap is converged within 50 meV for a grid density of $6 \times 6 \times 6$, while the high frequency dielectric constant requires a much denser sampling of $12 \times 12 \times 12$. A full GW calculation with this latter grid for all systems described in this manuscript would require extensive computational resources. Since the quasiparticle band gap is not as sensitive to the $\mathbf{k}$-grid density as the dielectric constant for these systems, we chose to use a sparser grid of $6 \times 6 \times 6$. As we will show in the next section, we will use the fully converged dielectric constants for the calculation of exciton binding energies. Furthermore, in all calculations we use a polarizability cutoff of 15 Ry and sum over 1000 bands. Overall, this setup achieves a converged quasiparticle correction within less than 0.1 eV for both $CsPbI_3$ and $CsPbBr_3$, as shown in Figure S1b-d.

Both DFT and quasiparticle band structures are interpolated using Wannier functions as implemented in the Wannier90 code.[12] Maximally localized Wannier functions are obtained starting from p orbitals on Pb and halogen atoms to generate an initial guess for the unitary transformations, for a total of 24 wannierized bands. The Wannier interpolated and DFT computed band structures agree within 1 meV in an energy window up to 2 eV above and below the valence band top. We then use the Bloch to Wannier rotation matrices to interpolate the quasiparticle band structures along path Γ (0, 0, 0) - X (0.5, 0, 0) - M

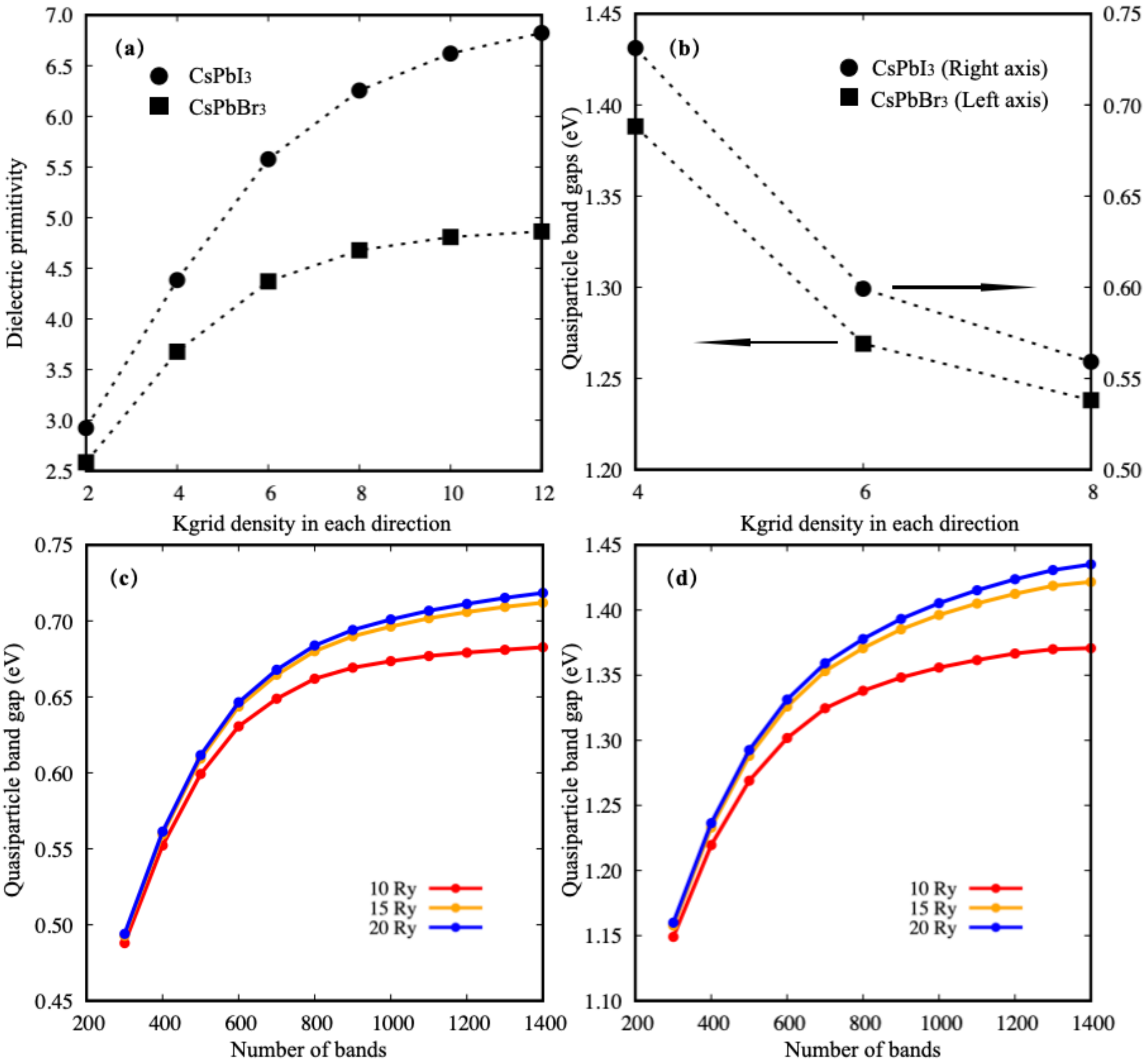

Figure S1: (a) Convergence of high frequency dielectric constants ε_∞ for $CsPbBr_3$ (squares) and $CsPbI_3$ (circles) with respect to **k**-point grid density. (b) Convergence of quasiparticle band gaps with respect to **k**-point grid density for $CsPbBr_3$ (squares) and $CsPbI_3$ (circles). (c,d) Convergence of quasiparticle band gaps with respect to the number of bands and polarizability cutoff for $CsPbI_3$ (c) and $CsPbBr_3$ (d), respectively.

(0.5, 0.5, 0) - Γ - R (0.5, 0.5, 0.5) - X from the quasiparticle eigenvalues on a 6×6×6 grid. We also tested the Wannier interpolated quasiparticle band structure against the discrete quasiparticle energies and found the same level of accuracy as in the DFT case (see Figure S2).

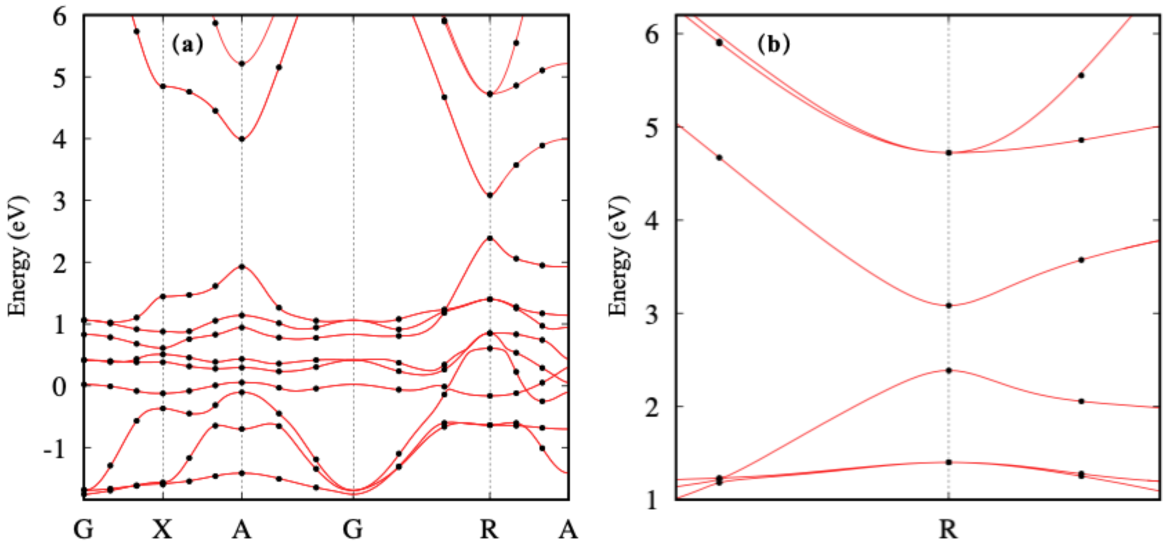

Figure S2: Wannier interpolated quasiparticle band structure (red lines) overlayed with discrete quasiparticle eigenvalues (black dots) calculated for $CsPbI_3$. The left panel shows a full path band structure as indicated in the text above, the right panel shows a zoom-in figure at R, to better visualize the interpolation near the band edges.

Charge-Carrier Effective Masses

Electron and hole effective masses m_e^* and m_h^*, are calculated from Wannier interpolated quasiparticle band structures with a parabolic band model. According to the $\mathbf{k} \cdot \mathbf{p}$ perturbation theory,[13] the effective mass should have a correlation with the band gap in a two-band isotropic model semiconductor, described as, $\frac{m_e}{m^*} = 1 + \frac{2}{m_e} \frac{|\mathbf{p}_{cv}|^2}{E_g}$, where m_e is the electron rest mass, m^* is the charge-carrier effective mass for electron and hole, E_g is the band gap, $\mathbf{p}_{cv}$ is the transition matrix element between the valence band top and conduction band bottom.[13] As shown in Figure 2c of the main manuscript, computed reduced effective masses follow very closely this simple model, and we can therefore use it to extrapolate effective masses with thermally corrected quasiparticle band gaps. Figure S3 shows by taking into account thermal correction, computed reduced effective masses yield better agreement with measurements for $CsPbBr_3$ and $CsPbI_3$, as reported in Ref. 14.

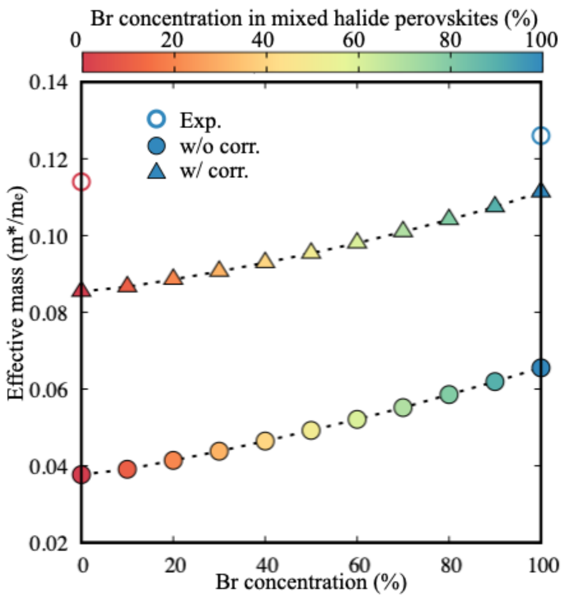

Figure S3: Reduced effective masses calculated from Wannier interpolated quasiparticle band structures (filled circles) and extrapolated effective masses with thermally corrected quasiparticle band gaps according to the $\mathbf{k}\cdot\mathbf{p}$ perturbation theory (filled triangles), compared with experimental values (empty circles) for $CsPbI_3$ and $CsPbBr_3$ reported in Ref.[14] All data points are color coded according to the concentration of Br, as shown in the color bar, and the dotted lines are guides to the eye.

Optical Excitations

We calculate the optical absorption spectra including electron-hole interactions by solving the Bethe-Salpeter equation (BSE) within the Tamm-Dancoff approximation[15,16] as implemented in BerkeleyGW package,[10] $\left(E^{\mathrm{QP}}_{c\mathbf{k}} - E^{\mathrm{QP}}_{v\mathbf{k}}\right) A^S_{vc\mathbf{k}} + \sum_{v'c'\mathbf{k}'} \left\langle vc\mathbf{k} \left| K^{\mathrm{eh}} \right| v'c'\mathbf{k}' \right\rangle A_{v'c'\mathbf{k}'} = \Omega^S A^S_{vc\mathbf{k}}$, where $A^S_{vc\mathbf{k}}$ are the coefficients of the exciton wavefunction in the quasiparticle basis, Ω^S is the corresponding excitation energy of state S, and K^{eh} is the electron-hole interaction kernel. The imaginary part of the dielectric function is calculated as,[10,16]

$$\varepsilon_2(\omega) = \frac{16\pi^2 e^2}{\omega^2} \sum_S |\mathbf{e} \cdot \langle 0|\mathbf{v}|S\rangle|^2 \delta\left(\omega - \Omega^S\right) \tag{1}$$

where we approximate the velocity operator as $\mathbf{v} = i\nabla$, as discussed in Ref. 10. We construct the electron-hole kernel using 10 valence bands and 10 conduction bands in a

coarse grid of 6×6×6 **k**-points, and then interpolate it to a fine grid of 40×40×40 **k**-points. We use 2 valence bands and 2 conduction bands (including spin degeneracy) in the fine grid to calculate the absorption spectrum. We calculate the absorption coefficient $\alpha(\omega)$ as $\alpha(\omega) = \frac{2\omega}{c}\sqrt{\frac{1}{2}\left(-\varepsilon_1(\omega) + \sqrt{\varepsilon_1^2(\omega) + \varepsilon_2^2(\omega)}\right)}$, and the exciton binding energy E_b is obtained directly by subtracting the optical band gap from the quasiparticle band gap.

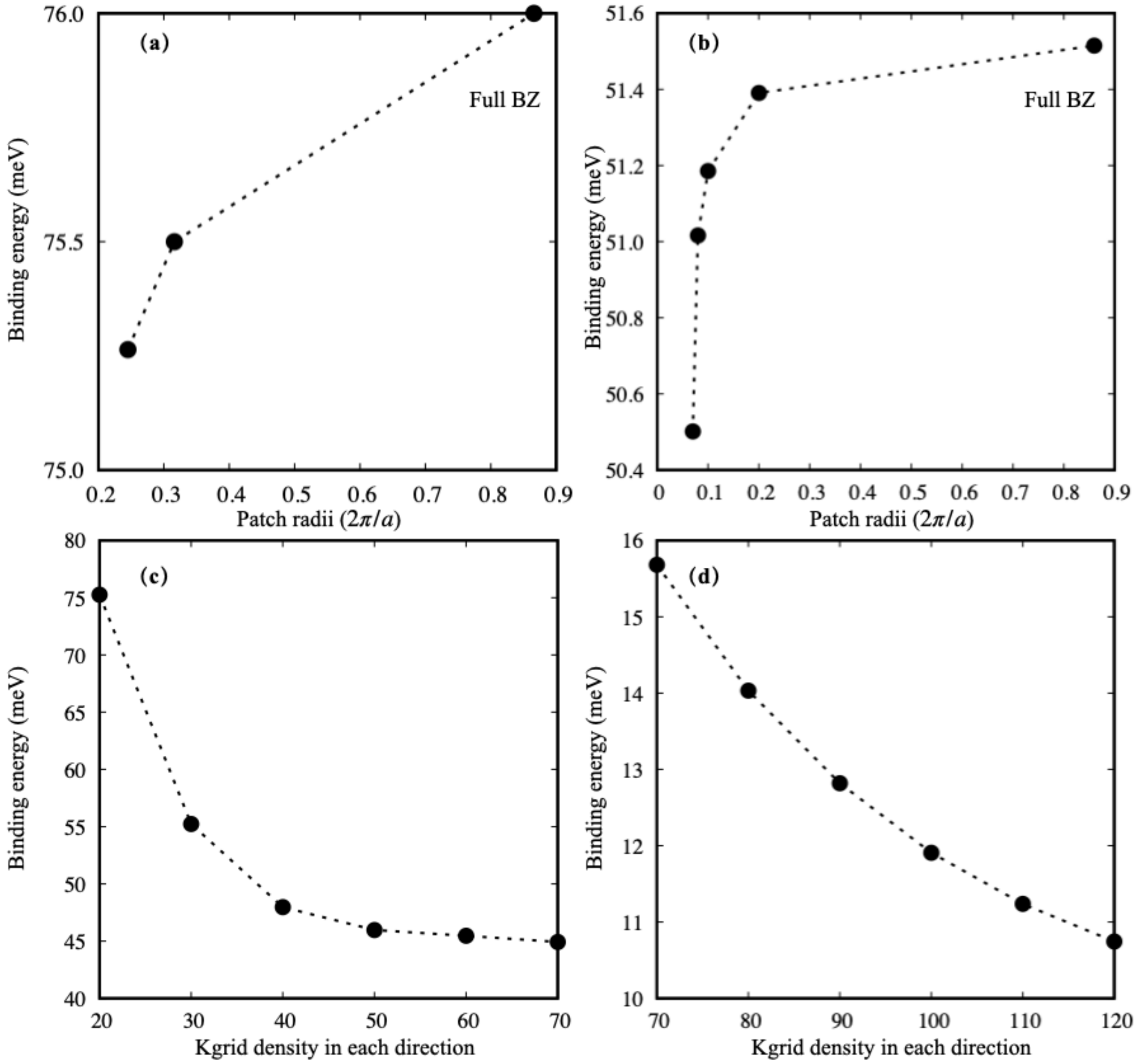

Figure S4: (a,b) Convergence of exciton binding energies with respect to patch size for (a) $CsPbBr_3$ and (b) $CsPbI_3$ (both tested using a $20 \times 20 \times 20$ **k**-point grid centered at Γ). The largest patch size corresponds to the full Brillouin zones in each case. (c,d) Convergence of exciton binding energies with respect to grid density for (c) $CsPbBr_3$ and (d) $CsPbI_3$ (calculated with converged patch radii of 0.25 $\times 2\pi/a$ and 0.07 $\times 2\pi/a$, respectively.)

To converge the exciton binding energy with respect to the density of the fine **k**-point mesh, we use the so-called 'patched sampling scheme',[17] which is based on the premise that a weakly bound exciton is highly localized in reciprocal space around the valence and conduction band edges. Following extensive convergence tests for $CsPbI_3$ and $CsPbBr_3$ (Figure S4), we find that the exciton binding energies are converged within 1 meV using patch radii of 0.07 $\times 2\pi/a$ for $CsPbI_3$ and $0.25\times 2\pi/a$ for $CsPbBr_3$, respectively, and densities equivalent to a uniform (Γ-centered) 120×120×120 grid for $CsPbI_3$ and 70×70 ×70 grid for $CsPbBr_3$, respectively. For our final converged calculations, we linearly interpolate the patch size (in unit of $2\pi/a$) and sampling grid density for all the mixed-halide compounds in the series $CsPb(Br_xI_{1-x})_3$.

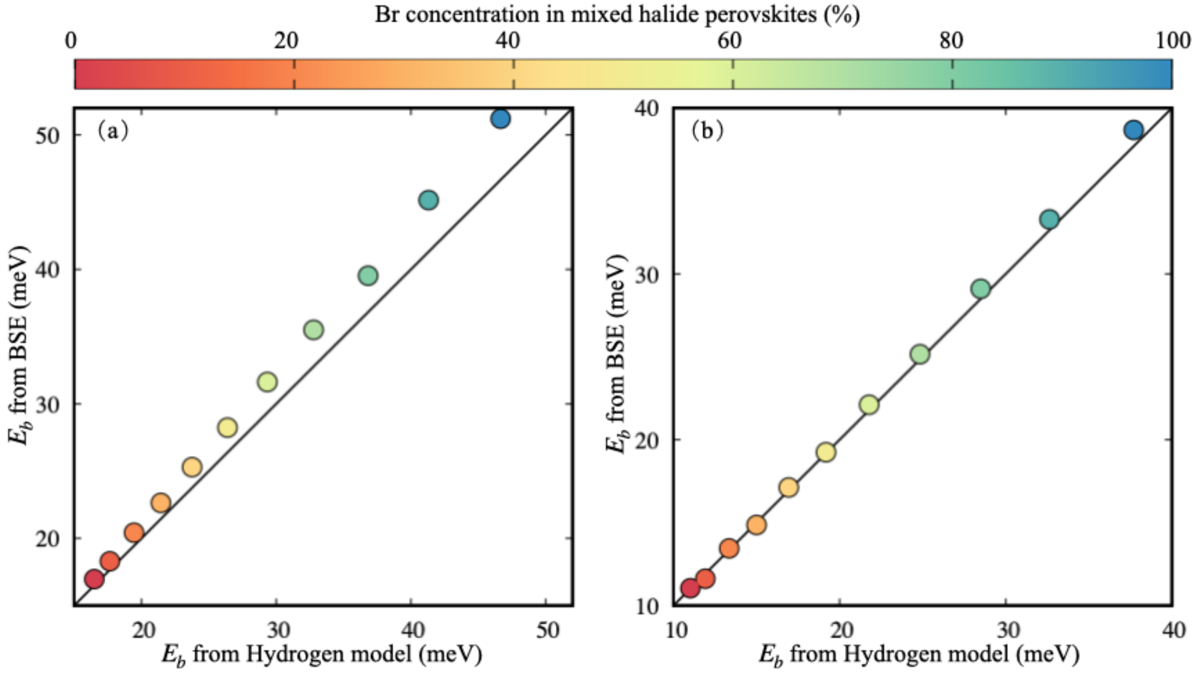

Figure S5: (a) Comparison between BSE calculated exciton binding energies with a 6×6×6 **k**-point grid and hydrogenic model exciton binding energies with the RPA ε_∞ calculated with the same grid. (b) Comparison between BSE calculated exciton binding energies using a dielectric function approximated as $\varepsilon(\mathbf{r}, \mathbf{r}') \sim \varepsilon_\infty$, with ε_∞ calculated from RPA with a 12×12×12 **k**-point grid and hydrogenic model exciton binding energies with the same ε_∞. All data points are color coded according to the concentration of Br, as shown in the color bar and the continuous black lines are the lines of perfect agreement between first principles and hydrogenic model.

In Figure S5a we show a comparison between exciton binding energies calculated from GW+BSE as described above and binding energies using the hydrogenic model,[18] $E_b = \mu^*/(m_e\varepsilon_\infty^2)$ Ry, where m_e is the electron rest mass, 1 Ry = 13.6057 eV is the Rydberg constant, and μ^* is the reduced effective mass from our quasiparticle band structure calculations. We find that *ab initio* exciton binding energies and hydrogenic model binding energies agree remarkably well, within a difference of at most 4 meV. This comparison indicates that the dielectric function can be approximated to be uniform throughout the crystal. We note that exciton binding energies calculated as described above are based on less converged RPA dielectric functions, as described in the previous section; according to convergence tests shown in Figure S1b, the dielectric constant for $CsPbI_3$ is underestimated by approximately 25% in this setup, and this is bound to impact the accuracy of calculated exciton binding energy. To improve on this, we use the observation made in Figure S5a, and recalculate the exciton binding energy from BSE, by approximating the dielectric function as $\varepsilon(\mathbf{r},\mathbf{r}') \sim \varepsilon_\infty$ (uniform screening approximation), where ε_∞ is the fully converged RPA dielectric constant calculated using a $12 \times 12 \times 12$ grid, as discussed in the previous section. For completeness, we also compare these exciton binding energies against the Wannier-Mott model and again obtain an excellent agreement. The exciton binding energies shown in Figure 3c of the main manuscript are calculated using the uniform screening approximation, using the best converged dielectric constant.

Calculated Charge-carrier Mobility Model

We calculate charge-carrier mobilities using a model proposed in Ref. 19, which assumes parabolic bands of effective mass m^* coupled to a single (highest energy) LO phonon with energy $\hbar\omega_{\mathrm{LO}}$, expressing the mobility μ_m as, $\dfrac{\mu_m}{e\hbar/m_e k_{\mathrm{B}}T} = \dfrac{0.052\,(\hbar\omega_{\mathrm{LO}}/k_{\mathrm{B}}T)^{3.3} + 0.34}{\alpha m^*/m_e}$.[19] α is the Fröhlich coupling constant given by $\alpha = \dfrac{e^2}{\hbar\epsilon_0}\left(\dfrac{m^*}{2\hbar\omega_{\mathrm{LO}}}\right)^{1/2}\left(\dfrac{1}{\varepsilon_\infty} - \dfrac{1}{\varepsilon_0}\right)$,[20] where ε_∞, ε_0 are the high- and low-frequency dielectric constants, and ϵ_0 is the vacuum permittivity.

All parameters in the expression of charge-carrier mobility are calculated in this work, and reported in Table S1.

Table S1: Parameters used to calculate the mobility μ_m: high-frequency (ε_∞) and static (ε_0) dielectric constants, the phonon energy of the principal LO mode ($\hbar\omega_{\mathrm{LO}}$), the corrected hole and electron effective masses (m_e^*/m_e, m_h^*/m_e) , the calculated Fröhlich coupling constant for hole and electron (α_h, α_e), the average electron and hole mobilities (μ_m^h, μ_m^e) and electron-hole sum mobility ($\mu_m^{\mathrm{sum}} = \mu_m^h + \mu_m^e$)

Br (%)	ε_∞	ε_0	$\hbar\omega_{\mathrm{LO}}$ (meV)	m_h^*/m_e	m_e^*/m_e	α_h	α_e	μ_m^h	μ_m^e	μ_m^{sum}
0	6.82	36.47	14.25	0.178	0.164	1.554	1.491	56.2	63.6	119.8
10	6.68	34.10	14.58	0.181	0.166	1.562	1.499	55.2	62.5	117.7
20	6.50	33.33	14.80	0.185	0.170	1.615	1.550	52.3	59.1	111.4
30	6.30	32.45	15.04	0.189	0.174	1.671	1.604	49.5	55.9	105.4
40	6.11	31.54	15.32	0.194	0.179	1.731	1.663	46.7	52.7	99.4
50	5.91	30.51	15.63	0.199	0.183	1.795	1.724	44.0	49.6	93.5
60	5.71	29.43	15.98	0.204	0.189	1.863	1.790	41.3	46.5	87.8
70	5.50	28.26	16.37	0.210	0.194	1.937	1.862	38.7	43.6	82.2
80	5.29	26.52	16.83	0.217	0.200	2.004	1.927	36.4	40.9	77.3
90	5.08	25.66	17.36	0.224	0.207	2.089	2.009	34.0	38.2	72.1
100	4.86	24.36	17.92	0.232	0.214	2.182	2.099	31.5	35.4	66.9

Experimental Methods

Sample preparation

All materials were used as purchased with no additional purification. All solvents, unless otherwise stated, were purchased from Sigma Aldrich. All precursor preparation and deposition has been performed in a N_2 filled glove box.

We used the following substrate cleaning procedure: z-cut quartz substrates were cleaned by subsequent sonication in Decon90 (1 vol% in deionised water), deionised water, acetone and isopropanol for 10 min each. After drying with a N_2 gun, the substrates were treated with UV-ozone for 15 mins.

$FA_{0.83}Cs_{0.17}Pb(Br_xI_{1-x})_3$ perovskite thin films were prepared as reported by Knight et al.[21] Briefly, $FA_{0.83}Cs_{0.17}PbI_3$ and $FA_{0.83}Cs_{0.17}PbBr_3$ were formed by weighing the precursors salts (formamidinium iodide, FAI, GreatCell Solar; cesium iodide, CsI, 99.99%, Alfa-Aesar; lead iodide, PbI_2, 99.999%, TCI; lead bromide, $PbBr_2$, >98%, Alfa-Aesar) were weighed to exact stoichiometry in an N_2 filled glovebox. The precursor salts were dissolved in a 4:1 ratio by volume of N,N-dimethylformamide (DMF, Sigma Aldrich) to dimethyl sulfoxide (DMSO, Sigma Aldrich), to a concentration of 0.7 M. The solutions were stirred overnight before deposition to form the intermediary Br composition, appropriate amounts of the solutions were mixed. Each solution was stirred for at least 30 mins prior to deposition.

The perovskite films were deposited on z-cut quartz substrates using the following spin-coating procedure:

Step 1: 50 μL of the precursor solution was deposited on to the quartz substrate which was spinning at 1000 rpm. After 5 s, the substrate accelerated a final spin speed of 5000 rpm over the course of 5 s.

Step 2: The substrate remained at 5000 rpm for 30 s. An antisolvent quench was performed by depositing 50 μl of Anisole 5 s before the end of this step. The films were then annealed at 100 °C for 30 min.

Transmission and reflection spectroscopy in the visible range

Reflection and transmission spectra were measured using a Bruker 80v Fourier-transform infra-red spectrometer with a tungsten halogen lamp source, a CaF_2 beamsplitter and a silicon diode detector. Data was collected with a resolution of 4 cm^{-1}. A silver mirror was used as reflection reference and a blank quartz substrate was used as transmission reference.

Terahertz spectroscopy

Time-domain terahertz spectroscopy (TDTS) and optical-pump terahertz-probe (OPTP) measurements were performed using a Spectra Physics Mai Tai-Empower-Spitfire Pro Ti:Sapphire regenerative amplifier. The amplifier generates 35 fs pulses centered at 800 nm at a repetition rate of 5 kHz. THz probe pulses were generated by a spintronic emitter which was composed of 1.8 nm of $Co_{40}Fe_{40}B_{20}$ sandwiched between 2 nm of Tungsten and 2 nm of Platinum, all supported by a quartz substrate. Detection of the THz pulses was performed using electro-optic sampling in a 1-mm (100)-ZnTe crystal. The sample, THz emitter and THz detector were held under vacuum ($<10^{-2}$ mbar) during the measurements. The optical pump excitation was obtained by frequency doubling the fundamental laser output through a BBO crystal, resulting in 400 nm pulses. The FWHMs of the beams for the pump and THz pulses at the sample are 2 mm and 0.6 mm respectively.

Modelling the Absorption Spectrum according to Elliott Formula

Elliott's theory describes the absorption of a semiconductor near the band edge.[22] Here, we follow the treatment described in Ref. 23, the total absorption coefficient α as a function of

incident energy E is given by

$$\alpha(E) = \frac{A_0}{E}\left(\sum_{n=1}^{\infty} \frac{4\pi E_b^{3/2}}{n^3}\delta(E-(E_g-\frac{E_b}{n^2})) + \frac{2\pi\sqrt{\frac{E_b}{E-E_g}}}{1-\exp(-2\pi\sqrt{\frac{E_b}{E-E_g}})}D(E)\right) \quad (2)$$

where the pre-factor A_0 is proportional to the transition dipole moment, E_b is the exciton binding energy, E_g is the band gap, $\delta(E)$ is the Dirac delta function, and $D(E)$ is proportional to the joint density of states. $D(E) = 0$ below the band gap energy and is given by $\sqrt{E - E_g}$ for $E > E_g$. Broadening due to electron-phonon interactions, local fluctuations in the stoichiometry of the material, and energetic disorder is represented by convolution of $\alpha(E)$ with a normal distribution $N(0, \Gamma^2)$ which has mean 0 and standard deviation Γ.

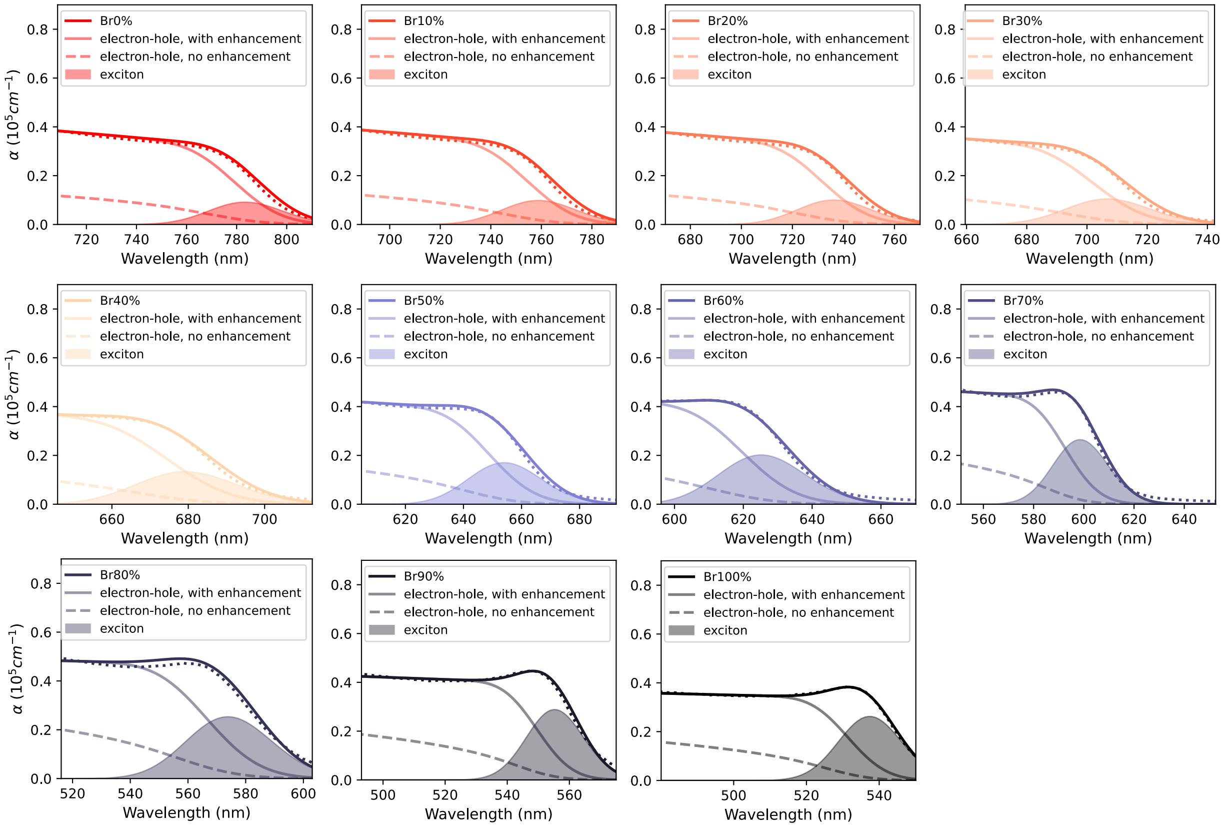

Figure S6: Absorption spectra (visible range) of $FA_{0.83}Cs_{0.17}Pb(Br_xI_{1-x})_3$. Dots are experimental data, dark solid lines are the Elliott fits, grey lines are the absorption of the continuum without Coulomb enhancement (see Ref. 23), and shaded area shows the contribution of the exciton resonance.

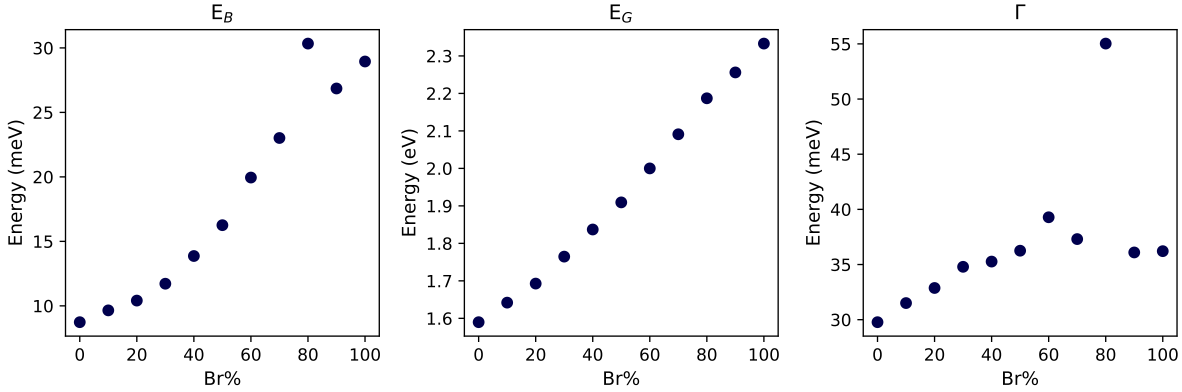

Figure S7: band gap, exciton binding energy and the standard deviation Γ of the normal distribution extracted from the Elliott fits to the absorption spectra of $FA_{0.83}Cs_{0.17}Pb(Br_xI_{1-x})_3$.

The absorption spectra of the $FA_{0.83}Cs_{0.17}Pb(Br_xI_{1-x})_3$ series were fitted with the resultant function. Figure S6 shows the spectra (experimental data as dotted lines and Elliott fit as solid lines), and the individual absorption associated with the exciton, as well as the absorption of the electron-hole continuum with and without the Coulomb enhancement, as described in Ref. 23. The fitted parameters (exciton binding energy, band gap and the standard deviation Γ of the normal distribution) are shown in Figure S7.

Determination of ε_∞

In order to determine ε_∞ from the reflectivity data, thin film interference effects must be taken into account. In addition to the Fabry-Perot interference effects, the data is also affected by a positive offset (likely associated with imperfections in the mirror reference used) and other uncertainties associated with scatter, which are more pronounced in the high Br content films. To fully take these factors into account, the experimental spectra were fitted to the modelled R. The modelled R is obtained from transfer matrix calculations for a sample comprising a semiconductor film of thickness d and refractive index r on a 2 mm quartz substrate. To take into account roughness and inaccuracies in the thickness of the films, d was allowed to vary within an interval ± 10 nm around the thickness values measured

using a Dektak profilometer. A small, wavelength-independent offset is also introduced to account for scatter and reference imperfections.

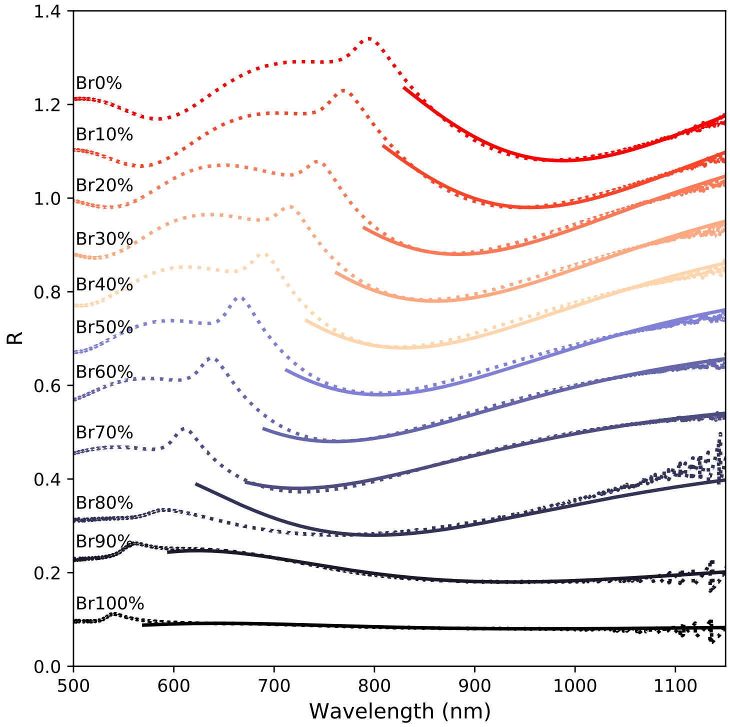

Figure S8: Reflectivity spectra of $FA_{0.83}Cs_{0.17}Pb(Br_xI_{1-x})_3$ films. An offset of 0.1 has been added between curves for clarity. Dotted lines are experimental data and solid lines are fits to a transfer matrix model.

Figure S8 shows the experimental reflectivity spectra (dotted lines) and the corresponding fits (solid lines). The values for the subgap refractive index obtained from the fits to the reflectivity spectra were used to calculate the high-frequency dielectric constant ε_∞, which are shown in Figure S9.

Determination of ε_0

The frequency-resolved THz spectra are obtained by varying the time delay between the THz pulse and a gate beam and performing a Fourier transform of the time-domain transmission of the THz radiation through the sample. The transmission through a blank quartz substrate is

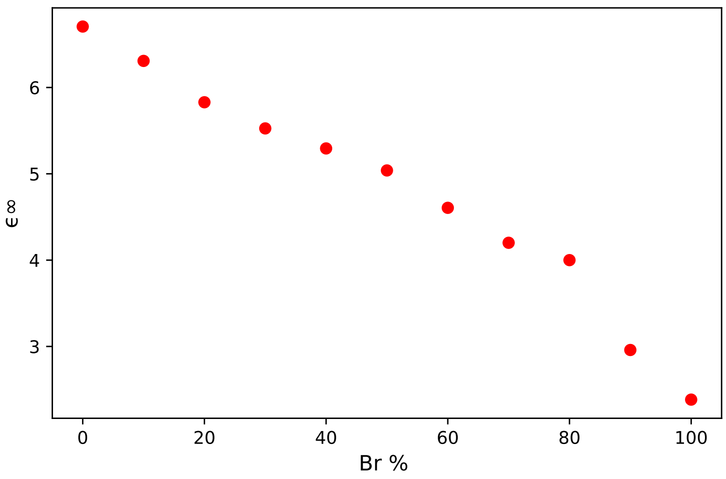

Figure S9: Values of ε_∞ for the various compositions of $FA_{0.83}Cs_{0.17}Pb(Br_xI_{1-x})_3$, calculated from the refractive index fitted to the subgap reflectivity spectra.

taken as a reference. The transmission function $T(\omega) = \frac{T_{sample}}{T_{substrate}}$ as a function of the angular frequency ω relates to the complex refractive index of the semiconductor film $\tilde{n}$ according to

$$T(\omega) = \frac{(1+\tilde{n}_s)}{(1+\tilde{n}_s) - i(\tilde{n}^2 + \tilde{n}_s)\omega d/c} \tag{3}$$

where $\tilde{n}_s$ is the substrate refractive index, and d is the thickness of the film.

The complex dielectric function $\varepsilon = \varepsilon' + i\varepsilon''$ can be determined from the experimental THz transmission spectra by substituting $\tilde{n}^2 = \varepsilon$.

The contribution of a phonon mode to the dielectric function can be described by a Lorentzian oscillator according to

$$\varepsilon_n(\omega) = A_n \frac{\omega_n^2}{\omega_n^2 - \omega^2 - i\omega\gamma_n} \tag{4}$$

where A_n is the amplitude of the contribution of the mode, which is proportional to the oscillator strength. ω_n is the resonance frequency and γ_n is the damping constant. In the present case of lead mixed halide perovskites, the experimental data can be successfully described as a combination of two TO modes. The total ε is then given by

$$\varepsilon(\omega) = \varepsilon_\infty + \varepsilon_{TO1} + \varepsilon_{TO2} \tag{5}$$

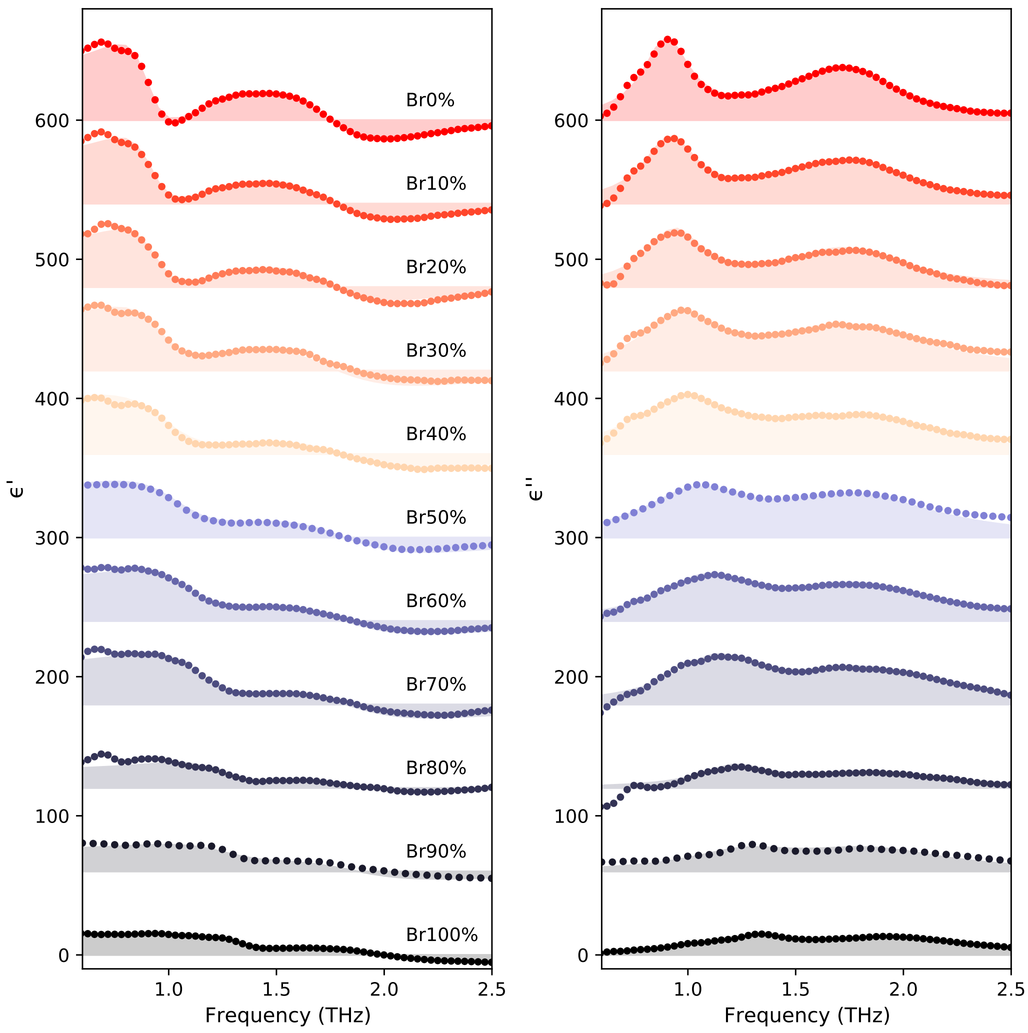

Figure S10: THz dielectric function of $FA_{0.83}Cs_{0.17}Pb(Br_xI_{1-x})_3$. Dots are experimental data, solid line and shaded area are fits to Equation 5.

The values of ε_∞ for the $FA_{0.83}Cs_{0.17}Pb(Br_xI_{1-x})_3$ series were obtained as described in the previous section. The THz spectra were then fitted to Equation 5. Figure S10 shows the experimentally measured THz spectra of the $FA_{0.83}Cs_{0.17}Pb(Br_xI_{1-x})_3$ films (dots) and the corresponding fits (solid lines and shaded area).

The resonance frequencies ω_n and damping constants γ_n of the phonon modes are optimised by the fitting routine. The values of ω_n obtained for the two modes as a function of the Br composition are shown in Figure S11. The THz quasi-static dielectric constant ε_0 at the low-frequency end is obtained from the fitted spectra. Figure S12 shows the values of ε_0 as

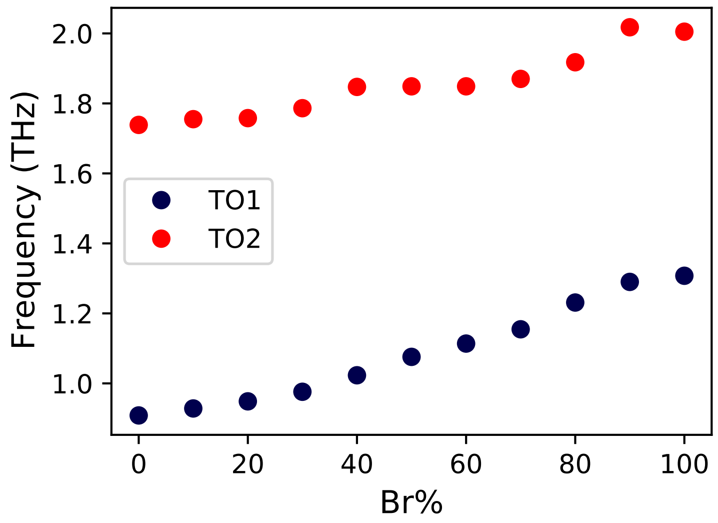

Figure S11: Resonance frequencies (THz) of the two TO phonon modes obtained from fits to the dielectric function of $FA_{0.83}Cs_{0.17}Pb(Br_xI_{1-x})_3$.

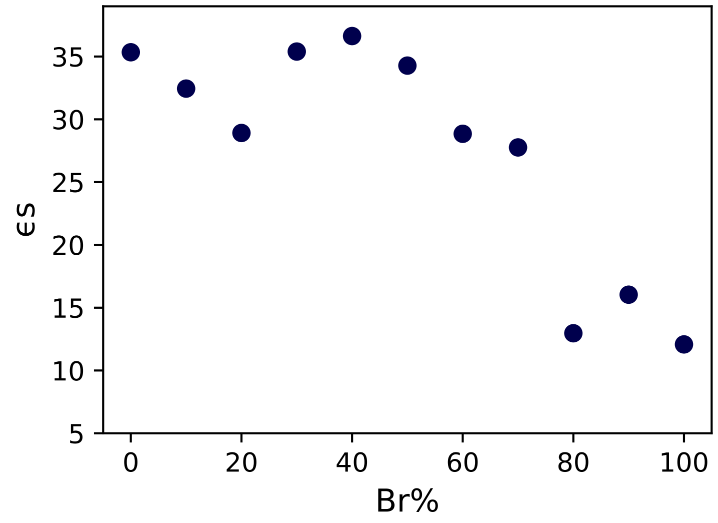

Figure S12: THz quasi-static dielectric constant ϵ_s obtained from fits to the THz dielectric function of $FA_{0.83}Cs_{0.17}Pb(Br_xI_{1-x})_3$.

a function of Br composition in the $FA_{0.83}Cs_{0.17}Pb(Br_xI_{1-x})_3$ series.

We note that the values obtained for both ε_∞ and ε_0 are likely affected by the uncertainties in the measurement of the semiconductor film thickness and optical density. These uncertainties can be associated with film roughness and also to material porosity. These factors, which are more pronounced in the high-Br content films, could lead to the overestimation of the

optical density and/or thickness and the underestimation of ε_∞ and ε_0.

Measured Charge-carrier Mobilities

The effective electron-hole sum mobility μ_m was extracted from the OPTP data using the method previously described by Wehrenfennig et al.[24] In brief, the sheet photo-conductivity, ΔS, of a material with a thickness much shorter than the wavelength of the THz radiation can be expressed as

$$\Delta S = -\epsilon_0 c(n_a + n_b)(\frac{\Delta T}{T}) \tag{6}$$

where c is the speed of light, ϵ_0 is the vacuum permittivity, and n_a and n_b are the THz refractive indices of the materials interfacing the perovskite layer at the front and rear respectively. The quantity $\Delta T/T$ is the ratio of the photo-induced change in THz electric field to the transmitted THz electric field in the dark. The initial number of photo-excited charge-carriers N is given by

$$N = \phi\frac{E\lambda}{hc}(1 - R_{\text{pump}} - T_{\text{pump}}) \tag{7}$$

with E being incident pump pulse energy, λ the excitation wavelength, ϕ the ratio of free charges created per photon absorbed, and R_{pump} and T_{pump} being the reflected and transmitted fractions of the pump beam. These two equations can be used to extract the charge-carrier mobility μ_m through

$$\mu_m = \frac{\Delta S A_{\text{eff}}}{Ne} \tag{8}$$

where A_{eff} is the effective area from the overlap of the pump and probe beams and e is the elementary charge. Substituting Equations 6 and 7 into Equation 8 we obtain

$$\phi\mu_m = -\frac{\epsilon_0 c(n_a + n_b)(A_{\text{eff}})}{Ne\lambda(1 - R_{\text{pump}} - T_{\text{pump}})}(\frac{\Delta T}{T}) \tag{9}$$

from which the effective charge-carrier mobility $\phi\mu_m$ may be determined based on the pump beam parameters and the initial measured $\Delta T/T$ of the sample. Here, μ_m is the charge-carrier mobility, and ϕ is the charge-to-photon branching ration which is assumed to be unity at room temperature. Charge-carrier mobility values were calculated based on the average of at least 10 measurements.

References

(1) Hohenberg, P.; Kohn, W. Inhomogeneous Electron Gas. *Phys. Rev.* **1964**, *136*, B864–B871.

(2) Garrity, K. F.; Bennett, J. W.; Rabe, K. M.; Vanderbilt, D. Pseudopotentials for High-Throughput DFT Calculations. *Comput. Mater. Sci.* **2014**, *81*, 446–452.

(3) Giannozzi, P. et al. Advanced Capabilities for Materials Modelling with Quantum ESPRESSO. *J. Phys.: Condens. Matter* **2017**, *29*, 465901.

(4) van Setten, M. J.; Giantomassi, M.; Bousquet, E.; Verstraete, M. J.; Hamann, D. R.; Gonze, X.; Rignanese, G. M. The PseudoDojo: Training and Grading a 85 Element Optimized Norm-Conserving Pseudopotential Table. *Comput. Phys. Commun.* **2018**, *226*, 39–54.

(5) Hamann, D. R. Optimized Norm-Conserving Vanderbilt Pseudopotentials. *Phys. Rev. B* **2013**, *88*, 085117.

(6) Bellaiche, L.; Vanderbilt, D. Virtual Crystal Approximation Revisited: Application to Dielectric and Piezoelectric Properties of Perovskites. *Phys. Rev. B* **2000**, *61*, 7877–7882.

(7) Baroni, S.; de Gironcoli, S.; Dal Corso, A.; Giannozzi, P. Phonons and Related Crystal

Properties from Density-Functional Perturbation Theory. *Rev. Mod. Phys.* **2001**, *73*, 515–562.

(8) Pérez-Osorio, M. A.; Milot, R. L.; Filip, M. R.; Patel, J. B.; Herz, L. M.; Johnston, M. B.; Giustino, F. Vibrational Properties of the Organic–Inorganic Halide Perovskite $CH_3NH_3PbI_3$ from Theory and Experiment: Factor Group Analysis, First-Principles Calculations, and Low-Temperature Infrared Spectra. *J. Phys. Chem. C* **2015**, *119*, 25703–25718.

(9) Hybertsen, M. S.; Louie, S. G. Electron Correlation in Semiconductors and Insulators: Band Gaps and Quasiparticle Energies. *Phys. Rev. B* **1986**, *34*, 5390–5413.

(10) Deslippe, J.; Samsonidze, G.; Strubbe, D. A.; Jain, M.; Cohen, M. L.; Louie, S. G. BerkeleyGW: A Massively Parallel Computer Package for the Calculation of the Quasiparticle and Optical Properties of Materials and Nanostructures. *Comput. Phys. Commun.* **2012**, *183*, 1269–1289.

(11) Godby, R. W.; Needs, R. J. Metal-Insulator Transition in Kohn-Sham Theory and Quasiparticle Theory. *Phys. Rev. Lett.* **1989**, *62*, 1169–1172.

(12) Pizzi, G. et al. Wannier90 as a Community Code: New Features and Applications. *J. Phys.: Condens. Matter* **2020**, *32*, 165902.

(13) Grosso, G.; Parravicini, G. P. In *Solid State Physics (Second Edition)*; Grosso, G., Parravicini, G. P., Eds.; Academic Press: Amsterdam, 2014; Chapter 2 - Geometrical Description of Crystals: Direct and Reciprocal Lattices, pp 67–105.

(14) Baranowski, M.; Plochocka, P.; Plochocka, P.; Su, R.; Legrand, L.; Barisien, T.; Bernardot, F.; Xiong, Q.; Testelin, C.; Chamarro, M. Exciton Binding Energy and Effective Mass of $CsPbCl_3$: A Magneto-Optical Study. *Photonics Res.* **2020**, *8*, A50–A55.

(15) Rohlfing, M.; Louie, S. G. Electron-Hole Excitations in Semiconductors and Insulators. *Phys. Rev. Lett.* **1998**, *81*, 2312–2315.

(16) Rohlfing, M.; Louie, S. G. Electron-Hole Excitations and Optical Spectra from First Principles. *Phys. Rev. B* **2000**, *62*, 4927–4944.

(17) da Jornada, F. H.; Qiu, D. Y.; Louie, S. G. Nonuniform Sampling Schemes of the Brillouin Zone for Many-Electron Perturbation-Theory Calculations in Reduced Dimensionality. *Phys. Rev. B* **2017**, *95*, 035109.

(18) Wannier, G. H. The Structure of Electronic Excitation Levels in Insulating Crystals. *Phys. Rev.* **1937**, *52*, 191–197.

(19) Poncé, S.; Schlipf, M.; Giustino, F. Origin of Low Carrier Mobilities in Halide Perovskites. *ACS Energy Lett.* **2019**, *4*, 456–463.

(20) Fröhlich, H. Electrons in Lattice Fields. *Adv. Phys.* **1954**, *3*, 325–361.

(21) Knight, A. J.; Borchert, J.; Oliver, R. D. J.; Patel, J. B.; Radaelli, P. G.; Snaith, H. J.; Johnston, M. B.; Herz, L. M. Halide Segregation in Mixed-Halide Perovskites: Influence of A-Site Cations. *ACS Energy Lett.* **2021**, *6*, 799–808.

(22) Elliott, R. J. Intensity of Optical Absorption by Excitons. *Phys. Rev.* **1957**, *108*, 1384–1389.

(23) Davies, C. L.; Filip, M. R.; Patel, J. B.; Crothers, T. W.; Verdi, C.; Wright, A. D.; Milot, R. L.; Giustino, F.; Johnston, M. B.; Herz, L. M. Bimolecular Recombination in Methylammonium Lead Triiodide Perovskite Is an Inverse Absorption Process. *Nat. Commun.* **2018**, *9*, 293.

(24) Wehrenfennig, C.; Eperon, G. E.; Johnston, M. B.; Snaith, H. J.; Herz, L. M. High Charge Carrier Mobilities and Lifetimes in Organolead Trihalide Perovskites. *Adv. Mater.* **2014**, *26*, 1584–1589.
